# Seasonal Influenza Vaccination among Saudi Children: Parental Barriers and Willingness to Vaccinate Their Children

**DOI:** 10.3390/ijerph16214226

**Published:** 2019-10-31

**Authors:** Abdullah Alolayan, Bdoor Almotairi, Shouq Alshammari, Malak Alhearri, Mohammed Alsuhaibani

**Affiliations:** 1 Department of Pediatrics, College of Medicine, Majmaah University, Majmaah 11952, Saudi Arabia; a.alolayan@mu.edu.sa; 2 Medical Students, College of Medicine, Qassim University, Qassim 51452, Saudi Arabia; bdoornaid@gmail.com (B.A.); shouqxalshammari@gmail.com (S.A.); malakalheari@gmail.com (M.A.); 3 Department of Pediatrics, College of Medicine, Qassim University, Qassim 51452, Saudi Arabia

**Keywords:** influenza vaccine, knowledge, attitude, parents, immunization, children, Saudi Arabia

## Abstract

Influenza is an acute respiratory infection. It is a contagious viral illness which can cause moderate to severe symptoms. However, high-risk groups, including children, can develop a severe condition requiring hospitalization that may, in severe cases, result in death. This study aimed to assess the knowledge and attitudes of Saudi parents toward the influenza vaccine and identify potential barriers to receiving the influenza vaccination. A cross-sectional survey was conducted using a questionnaire comprising 27 validated questions to assess parental awareness, knowledge, and attitudes toward the influenza vaccine. The overall attitude of the participants was positive (94.7%). However, their knowledge was generally poor (61.7%). Most participants were aware of the seasonal influenza vaccine (85.5%) and their children were up-to-date with the child national vaccination program vaccines (92.7%). Medical staff and awareness campaigns were the commonest sources of vaccine information. Significant predictors for knowledge about and attitudes toward the influenza vaccine included educational level, working in the medical field, monthly income, awareness of the seasonal influenza vaccine, having received the vaccine as parent, and having a child already vaccinated. Adherence to the influenza vaccination regimen for parents and their children was low. More educational campaigns are needed to increase knowledge about the vaccine.

## 1. Introduction

Influenza is an infectious disease that affects the upper and lower respiratory tracts and is caused by either the type A or B influenza virus. It is typically spread through coughing and sneezing, which cause the respiratory droplets of an infected individual to be aerosolized [1]. Influenza continuously circulates, causing seasonal or year-round epidemics according to the climate of the region. Seasonal influenza is a substantial health burden, and vaccination is the primary tool for prevention of influenza. However, changes in the predominant circulating strains from year-to-year viral mutations necessitate an annual modification of vaccine strains to match the currently circulating global influenza strains, and annual vaccination [2].

In Saudi Arabia, there is a uniquely challenging situation due to the pilgrimage season when millions of people, some carrying influenza virus, from different countries converge in the country. This is a common time for influenza outbreaks. The United States Advisory Committee on Immunization Practices recommends that everyone above 6 months of age have an annual influenza vaccination, unless there are medical contraindications. In United States, influenza vaccine coverage among children is low (57.9%) for a variety of reasons including feeling unlikely to become ill or having already been ill. Influenza-associated mortality is highest among children <2 years of age and in those with pre-existing conditions, although approximately half of the influenza mortality cases occur in healthy children [3,4,5,6]. The national immunization coverage among children in Saudi Arabia is above 90% [7]. Although the Saudi Ministry of Health and the Saudi Thoracic Society recommend the seasonal influenza vaccine annually for children above six months of age, the influenza vaccine coverage among Saudi children is unknown [8,9].

Parental knowledge about vaccines and the attitudes they have toward them influences whether or not their children are immunized [10]. One previous study showed that positive attitudes toward the influenza vaccine were associated with parental educational levels [11]. Doctors’ recommendations are another important factor that increases parental acceptance of the vaccine, and their role in expanding parents’ knowledge about the vaccine has been shown to be important in a majority of the studies investigating vaccine acceptance [12,13]. Furthermore, the media—the Internet, television, and magazines—have also been shown to have a strong impact on parental awareness of the influenza vaccine [14,15,16].

In Saudi Arabia, there are few studies that have investigated parental beliefs and barriers to administering the influenza vaccine to children [17,18]. Therefore, this study aimed to measure parents’ attitudes toward the influenza vaccine and to identify possible barriers toward having their children vaccinated against influenza. This was intended to shed light on parents’ decision-making processes, and to provide results to guide the development of recommendations that will increase seasonal influenza vaccination rates in children.

## 2. Materials and Methods

A cross-sectional study was conducted using a self-administered questionnaire that consisted of 29 questions designed to estimate parents’ awareness, knowledge, and attitudes about the influenza vaccine. The study population comprised Saudi nationals in different parts of the Qassim region who attended primary healthcare centers (PHCs) and had at least one child above six months of age at the time of the study. The Qassim region has a population of approximately 1.5 million, of which 80.4% are Saudi nationals. A multistage random sampling technique was used. In Stage 1, all PHCs in Buraydah, Unaizah, and Alrass—the three largest cities in the Qassim region, based on population size—were chosen.

During Stage 2, PHCs in each city were selected from an official list provided by the Ministry of Health, using a simple random sampling technique. In Stage 3, parents were chosen from each PHC using convenience sampling. The questionnaire used in the study was validated in two steps. First, it was revised by three faculty members with clinical and research experience. Second, it was tested in a pilot study using a sample of 20 parents who were not included in the present study. The questionnaire included four sections: Section 1, demographic data; Section 2, influenza vaccine awareness; Section 3, influenza vaccine knowledge; and Section 4, attitudes toward the influenza vaccine.

A consent form written in Arabic was attached to the questionnaire and filled out prior to answering the questionnaire. This study was approved by Qassim regional research ethics committee. The inclusion criteria were: being a Saudi national and being a parent with at least one child above six months of age. Non-Saudis, Saudis without children, and Saudi parents with children under six months of age, were excluded. The required sample size was estimated to be 385. The target sample size was increased to 400 during data collection to compensate for incomplete questionnaires.

Participants’ seasonal influenza vaccine knowledge scores were measured using nine questions. The most appropriate answer was identified and coded as 1; inappropriate or wrong answers were coded as 0. The knowledge score was calculated by summing all nine questions. The knowledge scores ranged from 0 to 9 and had been generated by using a cutoff point of 60% of the total score. Knowledge scores between 0 and 5 were classified as poor knowledge, while scores between 6 and 9 were classified as good knowledge.

Participants’ attitudes toward the seasonal influenza vaccine were evaluated using seven questions in a 5-point Likert scale format. The answer option “strongly disagree” was coded as 1, “disagree” as 2, “neutral” as 3, “agree” as 4, and “strongly agree” as 5. The attitude score was calculated by summing the scores of the responses to the seven questions, yielding a possible score range of 7–35. Scores in the range of 7–21 were classified as negative attitudes, and scores in the range of 22–35 were classified as positive attitudes. The reasons for parents not vaccinating their child were proposed by the research team and the faculty members who reviewed the questionnaire.

Descriptive statistics are presented using frequencies and proportions for all qualitative variables. The mean ± standard deviation was used for quantitative variables. Comparisons between dependent variables and independent factors were calculated using the Mann–Whitney U test and the Kruskal–Wallis test. The overall distribution of the data was measured using the Kolmogorov-Smirnov and Shapiro-Wilks test. A *p*-value <0.05 was considered significant. All data analyses were carried out using the Statistical Packages for Software Sciences (SPSS) version 21 (IBM Corporation, Armonk, NY, USA).

## 3. Results

In this study, 399 participants were recruited, giving a response rate of 99.7%. Most participants (69.2%) were mothers. The sociodemographic data are presented in Table 1.

The percentage of children who had received the seasonal influenza vaccine was 37.6%. Awareness about the influenza vaccine was relatively high (85.5%), with nearly all participants stating that their child was up-to-date with vaccinations provided by the national vaccination program. Approximately three out of four parents were aware that seasonal influenza vaccines were available in the hospitals and PHCs While most parents were aware of the influenza vaccine, less than a half of them (46.6%) had been vaccinated against influenza, and 63.4% of them had their child(ren) vaccinated against influenza. On the other hand, 53.3% of the parents did not receive the influenza vaccine and 85% did not vaccinate their children against influenza. 

Participants’ knowledge of the seasonal influenza vaccine is shown in Table 2. The commonest source of information was medical staff (29.3%), followed by awareness campaigns (25.2%), and the Internet (4.1%).

**Figure 1:**
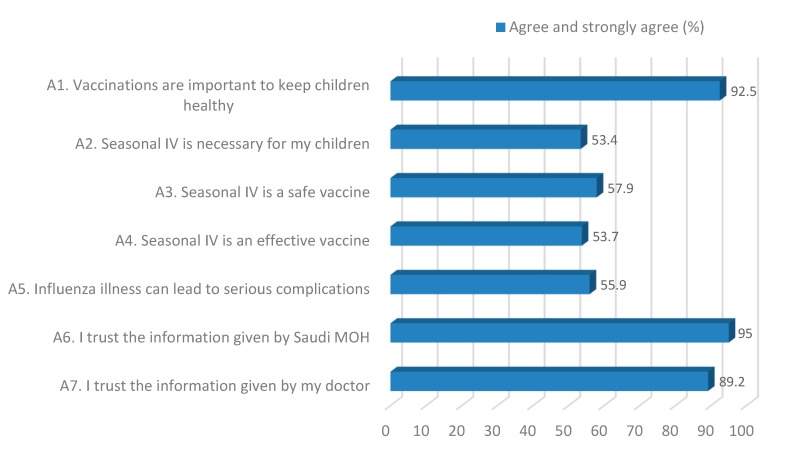
Attitudes and behaviors regarding the influenza vaccine among parents with at least one child aged six months or older (“strongly agree” and “agree” responses). Seasonal IV: Seasonal influenza vaccine; MOH: Ministry of Health.

The attitudes and behaviors of parents regarding the influenza vaccine is shown in Figure 1. The majority of the respondents either agreed or strongly agreed with each statement, with high agreement occurring for statements A1, A6, and A7.

**Figure 2:**
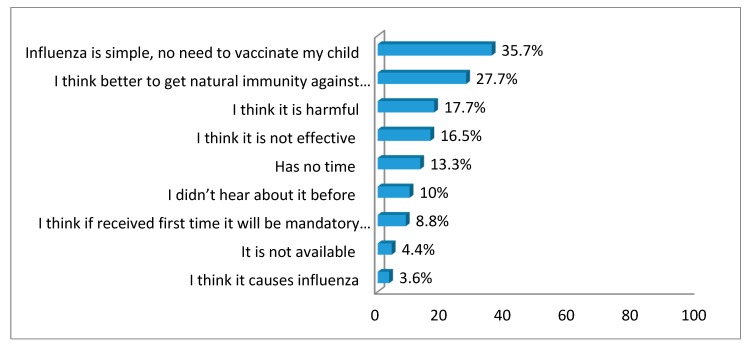
Reasons given by parents for not having their child(ren) vaccinated against influenza.

The commonest reasons given for not immunizing children against influenza was the belief that influenza is a simple illness that does not require vaccination, and the belief that natural immunity is better than vaccinations. A small percentage believed the statement “influenza vaccine causes influenza” (Figure 2).

The knowledge and attitude scores (Table 3) are shown according to the sociodemographic characteristics of the participants (Table 4). Men had a significantly higher level of knowledge than women, and a higher level of knowledge was also associated with a higher level of education, higher household income, and working in the medical field. Women had a significantly more positive attitude than men, and a positive attitude was also associated with a higher level of education, working in the medical field, and a low household income. Neither knowledge nor attitudes were associated with age, number of children, or city of residence. Parents who were more knowledgeable were significantly more likely to have heard of the influenza vaccine, been vaccinated against influenza, and to have a child who had been vaccinated against influenza. Parents with a positive attitude were significantly more likely to have been vaccinated against influenza, and to have a child who had been vaccinated against influenza.

## 4. Discussion

The present study investigated Saudi parents’ knowledge, attitudes, and practices toward the seasonal influenza vaccine. Our results show that while the attitude of parents toward vaccinations was generally positive, their overall knowledge regarding them was generally poor.

In this study, the majority of parents had heard of the seasonal influenza vaccine, and they were, for the most part, up to date with the child national vaccination program. In another study, Alabbad et al. [18] reported that 89% of participants in Riyadh, Saudi Arabia were aware of the influenza vaccine, which is similar to that found in our study. The previous study focused on both healthcare workers and parents, whereas this study focused only on parents.

The influenza vaccine coverage among children varies between countries; in Jordan 29% of children get the influenza vaccine, while influenza vaccine coverage among children in the United States is 57% [3,19]. However, in Pakistan only one-quarter of the parents (24.4%) were aware of the availability of influenza vaccine, and only 6.6% of parents vaccinated their children against influenza [11]. Furthermore, in Turkey, 27% of the parents reported that they received influenza vaccine during the H1N1epidemic, while 21.1% of their children received it [20]. In our study, three out of four parents knew that the seasonal influenza vaccine was available at either hospitals or PHCs, but less than half of the parents had been vaccinated against influenza, and less than 40% had their children vaccinated against influenza. Hence, the parents’ awareness about the availability of the vaccine and getting the vaccine for themselves are important factors in improving influenza vaccine coverage among children.

Agreement with the statements “influenza is simple, so there is no need to vaccinate my child” and “natural immunity is better than the influenza vaccine” were the commonest reasons parents gave for not getting the seasonal influenza vaccine for their child. The thought that the influenza vaccine could result in an influenza infection has been documented in responses from certain parents in our study. Besides, insufficient knowledge about the seriousness of the influenza and its consequences were demonstrated as well in Jordan [19]. In other studies, reasons for refusing seasonal influenza vaccination varied from region to region and included lack of a doctor’s recommendation, worries about side effects, and doubts regarding the efficacy of the vaccine [12,19,21]. Therefore, providing information on the incidence of influenza to the general public via the media and awareness campaigns may affect beliefs toward influenza. Moreover, parents should be educated on the safety of the vaccine to correct misconceptions.

In our study, the most reliable sources of vaccine information came from medical staff and awareness campaigns, which is in agreement with other studies (18–21). This suggests that physicians have a critical role to play in optimizing parents’ knowledge about influenza and the influenza vaccine. Therefore, further educating physicians about the importance and risks of influenza should be considered by the Ministry of Health and hospital directors.

Most parents in this study held positive attitudes and exhibited positive behaviors regarding vaccinations while the negative attitudes were secondary to lack of knowledge about the safety and effectiveness of the influenza vaccine. Besides, one study in Riyadh found that confidence in the Saudi Ministry of Health among healthcare workers was relatively high, with 90% of them also trusting the information coming from their physicians [18]. In Jordan, most of the parents had a positive attitude toward the influenza vaccine when they received information about its protective role and benefits [19].

We also found that women held stronger positive attitudes than men toward the influenza vaccine. Participants that worked in the medical field also exhibited better knowledge and attitudes, which is an important factor in supporting and disseminating accurate information regarding the vaccine in the Saudi community. However, working in the medical field does not warrant good knowledge and/or positive attitude toward the influenza vaccine, as reported in some regional studies [22,23].

Additionally, Awad et al [19] found a significant relationship between child vaccination and parents’ vaccination status, as the majority of parents who were vaccinated against influenza had their children vaccinated. We observed the same relationship in this study; thus, optimizing the knowledge and attitude toward the influenza vaccine among the parents would certainly reflect positively in the vaccination status of their children. Furthermore, participants who were aware of the seasonal influenza vaccine had better knowledge, and those who received the influenza vaccine or had vaccinated their child were associated with having good knowledge and positive attitudes toward the vaccine.

This study has some limitations. Specifically, the study took place in a single region and may not be generalizable to other regions of Saudi Arabia. Additionally, the convenience sampling method may have introduced sampling bias.

Despite these limitations, our overall results shed light on parental perspectives on the seasonal influenza vaccine in Saudi Arabia and highlight the need to increase parental awareness on the importance of seasonal influenza vaccination. Additionally, as the study’s sample size is reasonably large, our results can supplement other findings related to seasonal influenza vaccination.

## 5. Conclusions

In Saudi Arabia, parental attitudes toward the seasonal influenza vaccine was generally positive; however, knowledge was generally poor. Most parents were aware of the seasonal influenza vaccine, but their adherence to receiving the vaccination for both themselves and their children was found to be low. Educational level, working in the medical field, monthly income, awareness about the vaccine, having received the influenza vaccine as a parent, and having given their child the influenza vaccine were all significant factors associated with both vaccine knowledge and attitudes. More awareness campaigns and education by pediatricians or primary care doctors are needed to increase parental knowledge of the seasonal influenza vaccine. The importance of seasonal influenza vaccine and the potential health consequences of refusal to vaccinate should be highlighted in mass media and educational campaigns. 

## Figures and Tables

**Table 1:** Sociodemographic characteristics of participants.

Study Variables	*n* (%) (*N* = 399)
*Gender*	
Women	276 (69.2)
*Age group in years*	
18–24	32 (8.0)
25–34	140 (35.1)
35–44	128 (32.1)
45–54	68 (17.0)
55–65	31 (7.8)
*Educational level*	
Less than high school	69 (17.3)
High school graduate	117 (29.3)
College graduate	203 (50.9)
Master’s or PhD	10 (2.5)
*Working in a medical field*	
Yes	62 (15.5)
No	337 (84.5)
*Household monthly income (Saudi Riyal)*	
2000–6000	42 (10.5)
6001–10,000	49 (12.3)
10,001–15,000	72 (18.0)
>15,000	26 (6.5)
Did not know/refused	210 (52.6)
*Number of children*	
One	116 (29.1)
Two	100 (25.1)
Three	82 (20.6)
Four or more	101 (25.3)
*City of residence*	
Alrass	162 (40.6)
Buraydah	108 (27.1)
Unayzah	129 (32.3)

**Table 2:** Parental knowledge regarding the seasonal influenza vaccine.

Statement	Correct Answer *n* (%) (*N* = 399)
K1. Influenza is a common disease	351 (88.0)
K2. Influenza is highly contagious	291 (72.9)
K3. Influenza is transmitted through coughing and sneezing	372 (93.2)
K4. Influenza can lead to hospitalization and death	214 (53.6)
K5. The influenza vaccine is recommended for all children >6 months	156 (39.1)
K6. The aim of the influenza vaccine is to prevent influenza	115 (28.8)
K7. The influenza vaccine will not cause influenza	162 (40.6)
K8. The influenza vaccine should be given every year	227 (56.9)
K9. Antibiotics cannot treat a viral influenza infection	76 (19.0)

**Table 3:** Parental knowledge and attitudes toward the seasonal influenza vaccine.

Predictor	*n* (%) (*N* = 399)
Knowledge Score (mean ± SD)	04.9 ± 01.7
*Knowledge level*	
Poor	246 (61.7)
Good	153 (38.3)
Attitude Score (mean ± SD)	28.3 ± 04.3
*Attitude level*	
Negative	21 (5.3)
Positive	378 (94.7)

**Table 4:** Knowledge and attitude scores according to participant characteristics (*N* = 399).

Factor	Knowledge Total Score (out of 9) Mean ± SD	*p*-Value	Attitude Total Score (out of 35) Mean ± SD	*p*-Value
*Gender* ^a^				
Men	05.1 ± 01.8	0.448	27.1 ± 04.8	0.003 **
Women	04.9 ± 01.7		28.8 ± 03.9	
*Age group in years* ^b^				
18–34 years	04.8 ± 01.8	0.155	28.7 ± 04.2	0.284
35–44 years	05.1 ± 01.8		27.9 ± 04.1	
>44 years	04.8 ± 01.6		27.9 ± 04.6	
*Educational level* ^a^				
High school or below	04.5 ± 01.8	<0.001 **	27.7 ± 04.7	0.020 **
College graduate or higher	05.2 ± 01.6		28.8 ± 03.8	
*Working in medical field* ^a^				
Yes	05.9 ± 01.5	<0.001 **	29.2 ± 04.4	0.020 **
No	04.7 ± 01.7		28.1 ± 04.2	
*Household monthly income (SAR)* ^b^				
≤10,000	04.7 ± 01.8	<0.001 **	29.1 ± 04.5	0.032 **
>10,000	05.5 ± 01.8		28.6 ± 03.9	
Refused	04.7 ± 01.6		27.8 ± 04.3	
*Number of children* ^b^				
One	04.7 ± 01.6	0.120	28.3 ± 03.6	0.551
Two	04.8 ± 01.7		27.7 ± 04.6	
Three	04.9 ± 01.7		28.6 ± 04.1	
Four or more	05.2 ± 01.8		28.4 ± 04.8	
*City of residence* ^b^				
Alrass	04.9 ± 01.7	0.808	27.8 ± 04.9	0.616
Buraydah	04.9 ± 01.8		28.5 ± 03.7	
Unayzah	04.9 ± 01.7		28.6 ± 03.8	
*Heard about seasonal vaccine* ^a^				
Yes	05.2 ± 01.6	<0.001 **	28.4 ± 04.2	0.073
No	03.5 ± 01.6		27.4 ± 04.5	
*Received influenza vaccine as a parent* ^a^				
Yes	05.7 ± 01.6	<0.001 **	29.5 ± 04.1	<0.001 **
No	04.3 ± 01.5		27.2 ± 04.1	
*Child received influenza vaccine* ^a^				
Yes	05.7 ± 01.6	<0.001 **	30.5 ± 03.3	<0.001 **
No	04.4 ± 01.6		26.9 ± 04.2	

^a^*p*-value calculated using the Mann–Whitney U test. ^b^*p*-value calculated using the Kruskal–Wallis test. ** Significant at the *p* ≤ 0.05 level.
